# Better pathologic complete response and relapse-free survival after carboplatin plus paclitaxel compared with epirubicin plus paclitaxel as neoadjuvant chemotherapy for locally advanced triple-negative breast cancer: a randomized phase 2 trial

**DOI:** 10.18632/oncotarget.10607

**Published:** 2016-07-14

**Authors:** Pin Zhang, Yi Yin, Hongnan Mo, Bailin Zhang, Xiang Wang, Qing Li, Peng Yuan, Jiayu Wang, Shan Zheng, Ruigang Cai, Fei Ma, Yin Fan, Binghe Xu

**Affiliations:** ^1^ Department of Medical Oncology, Cancer Hospital, Chinese Academy of Medical Sciences and Peking Union Medical College, Beijing, China; ^2^ Department of Medical Oncology, Fujian Provincial Cancer Hospital, Fuzhou, China; ^3^ Department of Breast Surgery, Cancer Hospital, Chinese Academy of Medical Sciences and Peking Union Medical College, Beijing, China; ^4^ Department of Pathology, Cancer Hospital, Chinese Academy of Medical Sciences and Peking Union Medical College, Beijing, China

**Keywords:** breast cancer, triple negative, neoadjuvant chemotherapy, carboplatin, paclitaxel

## Abstract

Background: No standard chemotherapy is used as neoadjuvant therapy in triple negative breast cancer (TNBC). This study has compared carboplatin plus paclitaxel with commonly used epirubicin plus paclitaxel as neoadjuvant chemotherapy (NAC) in TNBC.

Results: 91 patients with a median age of 47 years (PC 47 patients, EP 44 patients) were enrolled. 65% of the patients were premenopausal. While the objective response rate was similar in the PC and EP arm (89.4% vs. 79.5%, *P* = 0.195), the pCR rate in the PC arm was significantly higher (38.6% vs. 14.0%, *P* = 0.014). The median follow-up time was 55.0 months. 5-year RFS were 77.6% and 56.2%, significantly higher in the PC arm, *P* = 0.043. No significant difference in OS was observed between the two arms (*P* = 0.350). Adverse events were similar, except for more thrombocytopenia in the PC arm (*P* = 0.001).

Methods: Patients with stage II/III TNBC were randomized to receive either paclitaxel (175 mg/m^2^, day1) plus carboplatin (Area Under the Curve = 5, day2) (PC) or epirubicin (75mg/m^2^, day1) plus paclitaxel (175 mg/m^2^, day2) (EP) as NAC every three weeks for 4-6 cycles. The primary endpoint was rate of pathologic complete response (pCR).The secondary endpoints included relapse-free survival (RFS), overall survival (OS) and safety.

Conclusions:This study suggested that the addition of carboplatin to paclitaxel was superior to the regimen of epirubicin plus paclitaxel as NAC for TNBC in terms of improving pCR rate and RFS. Further phase 3 study has already started.

## INTRODUCTION

Triple-negative breast cancer (TNBC), defined as the lack of expression of estrogen receptor (ER), progesterone receptor (PR), and human epidermal growth factor receptor 2 (HER2), accounts for approximately 20% of all breast cancers [1, 2]. Without specific treatment such as endocrine therapy or anti-HER2 therapy for this subtype of breast cancer, cytotoxic chemotherapy remains the only choice of treatment [2].

Neoadjuvant chemotherapy (NAC) with both anthracycline and taxane combination resulted in higher pathologic complete response (pCR) rate (28%) than those of anthracycline-based (20%) or taxane-based (12%) regimens [3]. It is believed to be the most effective regimen for TNBC in neoadjuvant setting. A pCR rate of 38.9% was observed after anthracycline-taxane-based NAC in patients with operable or locally advanced TNBC [4]. Although there was strong association between higher pCR rate and improved relapse-free survival (RFS) and overall survival (OS), the overall prognosis of TNBC remains poor including high risk of early recurrence involving viscera or central nervous system [1, 5, 6]. Data from M.D. Anderson Cancer Center showed that the 5-year RFS and OS rates were 61% and 64% after NAC in patients with operable or locally advanced TNBC [3]. The 7-year RFS and OS rates were only 57% and 65% in locally advanced TNBC after anthracycline-taxane-cyclophosphamide NAC [7].

The role of platinum-based NAC has been investigated in TNBC and was gradually brought to attention during the past few years. Results from two randomized phase II studies suggested that in TNBC, the addition of carboplatin to anthracycline-taxane-based NAC significantly improved the pCR rate, but resulted in more toxicity-related delay of treatment [8, 9]. Unfortunately, neither study has reported relapse-free survival and overall survival due to lack of long term follow up. Hurly et al retrospectively analyzed docetaxel plus carboplatin as anthracycline-free NAC in 27 locally advanced TNBC patients resulting in a pCR rate of 26% [10]. Shindle et al retrospectively reported a pCR rate of 60% and a 25-months of median relapse-free survival in 10 locally advanced TNBC patients treated by paclitaxel plus carboplatin NAC. [11].

To date, no prospective or randomized study has compared a platinum-based regimen to commonly used anthracycline-taxane regimens in TNBC in neoadjuvant setting. This randomized controlled phase II study is designed to compare paclitaxel plus carboplatin given as NAC with epirubicin plus paclitaxel in locally advanced TNBC.

## RESULTS

### Patient characteristics

**Figure 1:**
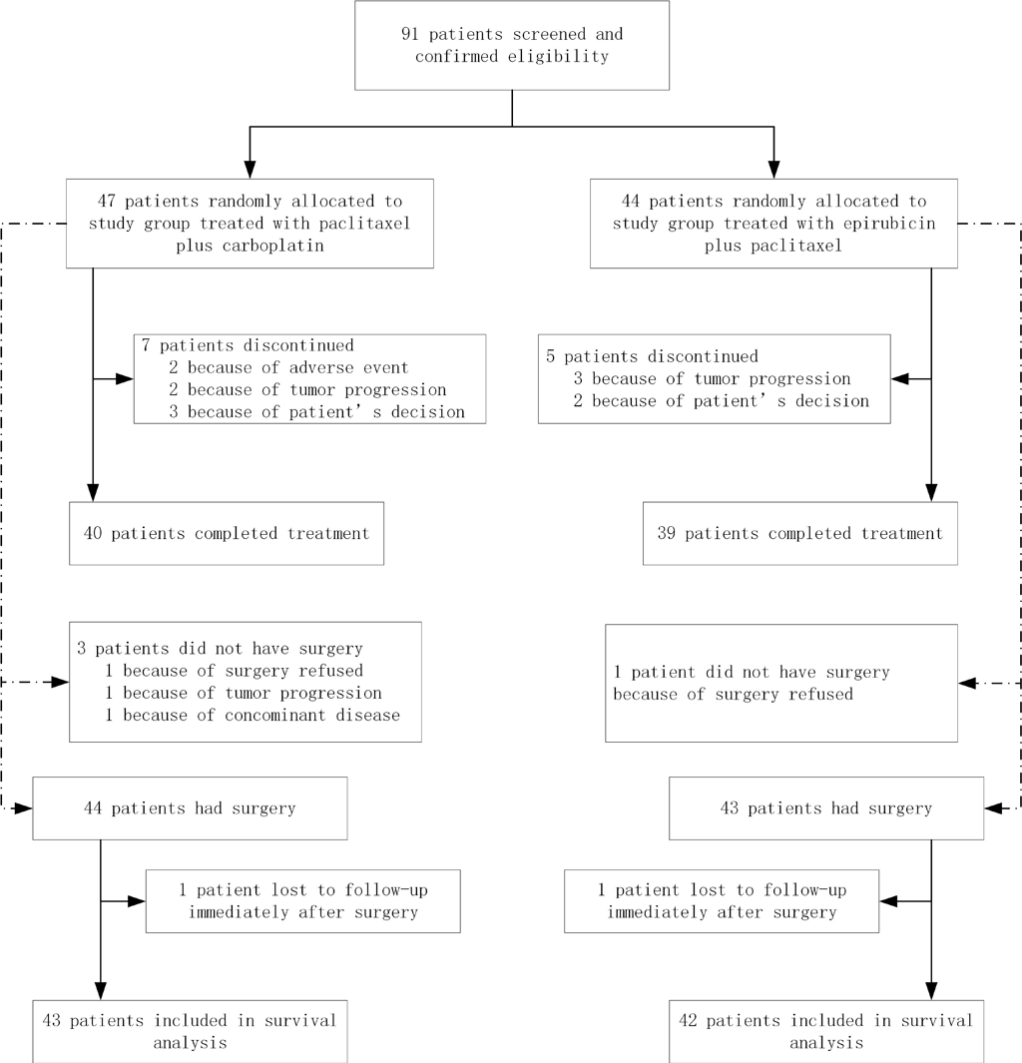
Trial profile

Between May 2006 and December 2012, 91 patients were enrolled and started study treatment. 47 patients with PC regimen and 44 patients with EP regimen were included in the intention-to-treat and safety populations (Figure 1).

Baseline characteristics of the 91 treated patients are listed in Table 1. Median age was 47 years (range 24-73 years). All enrolled patients were female, 65% of whom were premenopausal. More than half had T2 tumors. 76.9% of all patients were clinically node positive. More than two-thirds of patients had Ki-67 proliferation index > 20%. Notably, 94.4% of patients were positive for either CK5/6 or EGFR, 97.30% in PC arm and 91.67% in the EP arm, respectively (*P* = 0.358). Baseline characteristics were well balanced between two arms.

87 patients underwent surgery and had tumor responses evaluated pathologically. 75 patients underwent modified radical mastectomy and 12 patients had breast-conserving surgery. 79 patients received at least 4 cycles of NAC, and 38 patients completed six cycles of NAC. 4 patients in PC arm switched to anthracycline-based adjuvant chemotherapy, and 13 patients in EP arm switched to platinum-based regimen after surgery due to lack of objective response in NAC. 23 patients in the PC arm and 21 patients in the EP arm received post-operative radiotherapy. All the patients who underwent breast-conserving surgery received radiotherapy.

**Table 1:** Patient characteristics at baseline (intention-to-treat population)

Characteristics	PC arm (*N*= 47)	EP arm (*N* = 44)	*P* value
Age, median years (range)	48 (24–73)	46 (24–65)	0.205
Menopausal status			0.518
Premenopausal	29 (61.7%)	30 (68.2%)	
Postmenopausal	18 (38.3%)	14 (31.8%)	
Clinical tumor stage			0.271
T1	1 (2.13%)	4 (9.09%)	
T2	24 (51.6%)	25 (56.82%)	
T3	13 (27.66%)	16 (36.36%)	
T4	9 (19.15%)	6 (13.64%)	
Clinical nodal stage			0.055
N0	13 (27.66%)	8 (18.18%)	
N1	13 (27.66%)	21 (47.73%)	
N2	17 (36.17%)	7 (15.91%)	
N3	4 (8.51%)	8 (18.18%)	
Clinical stage			1.000
II	16 (34.04%)	15 (34.09%)	
III	31 (65.96%)	29 (65.91%)	
Ki-67			0.054
<20%	5 (13.16%)	12 (33.33%)	
>20%	33 (86.84%)	24 (66.67%)	
CK5/6, EGFR			0.358
Either positive	36(97.30%)	33(91.67%)	
Both negative	1(2.70%)	3(8.33%)	

Abbreviation: PC, paclitaxel plus carboplatin regimen; EP, epirubicin plus paclitaxel regimen; CK5/6, Cytokeratin 5/6; EGFR, epidermal growth factor receptor.

Data are n (%) unless stated otherwise.

### Clinical efficacy

**Figure 2:**
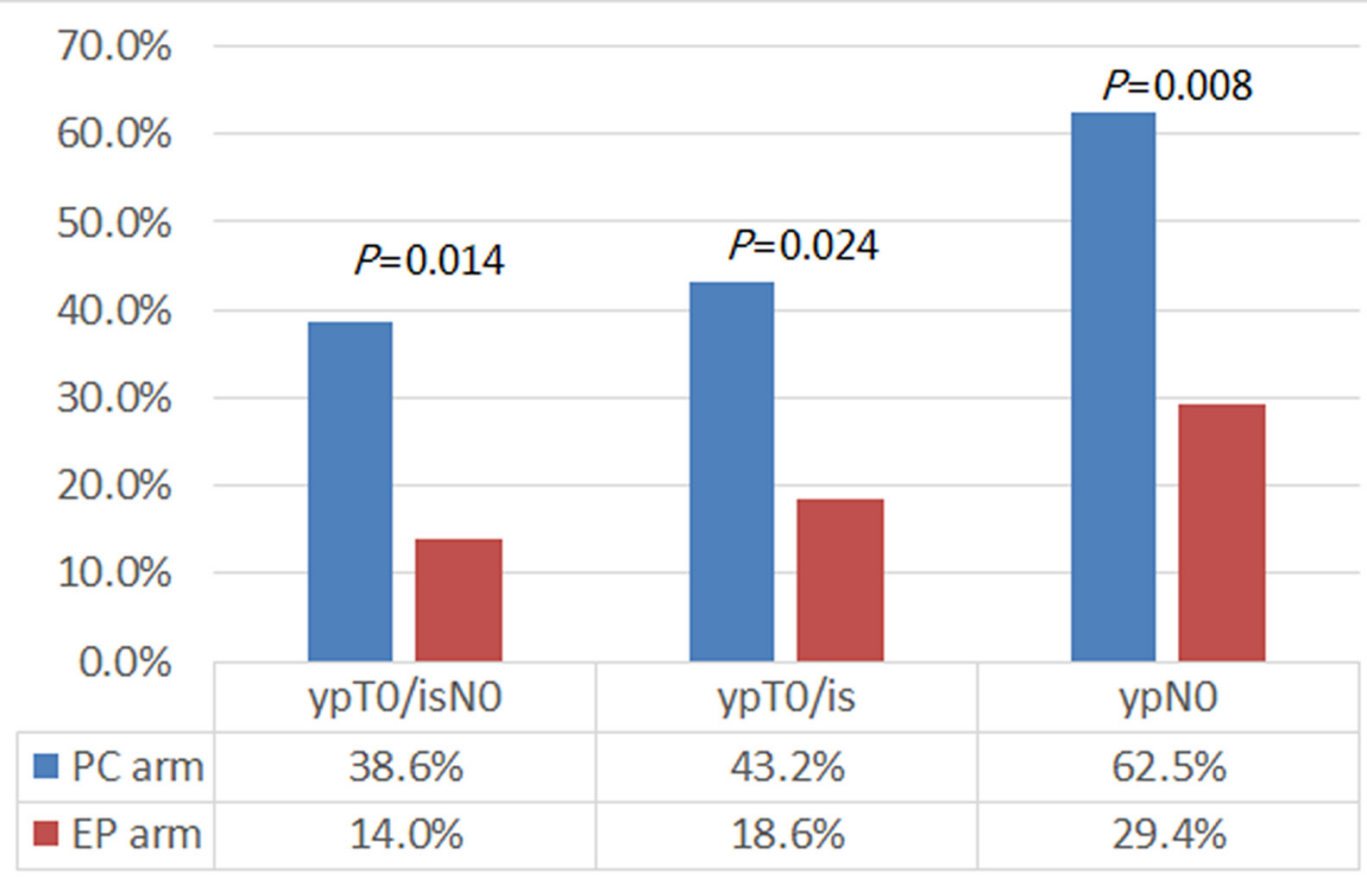
The pathologic complete response (pCR) rate of patients in different arms When compared with those in the EP arm, patients in the PC arm had significantly higher pCR (ypT0/isN0) rate (38.6% *vs*. 14.0%; *P* = 0.014), both in breast (ypT0/is; 43.2% *vs*. 18.60%; *P* = 0.024) and in axilla (ypN0; 62.5% *vs*. 29.4%; *P* = 0.008).

Of the 91 patients who were evaluated for clinical response, the overall ORR was similar between the two regimens (*P* = 0.195): 89.4% in PC arm and 79.5% in EP arm, respectively. 87 patients underwent surgery and had tumor responses evaluated pathologically. 23 of 87 patients (26.4%) had pCR (ypT0/isN0) of the invasive breast cancer both in breast and axilla, including 17 patients in PC arm and 6 patients in EP arm. The pCR (ypT0/isN0) rate in the PC arm was significantly higher compared to the EP arm (38.6% *vs*. 14.0%; risk ratio = 3.876; *P* = 0.014; Figure 2). Furthermore, 18/44 patients (43.2%) in PC arm and 8/43 patients (18.60%) in EP arm had pCR (ypT0/is) in breast (*P* = 0.024), whereas 20/32 patients (62.5%) in PC arm and 10/34 patients (29.4%) in EP arm had pCR (ypN0) in axilla (*P* = 0.008).

**Figure 3:**
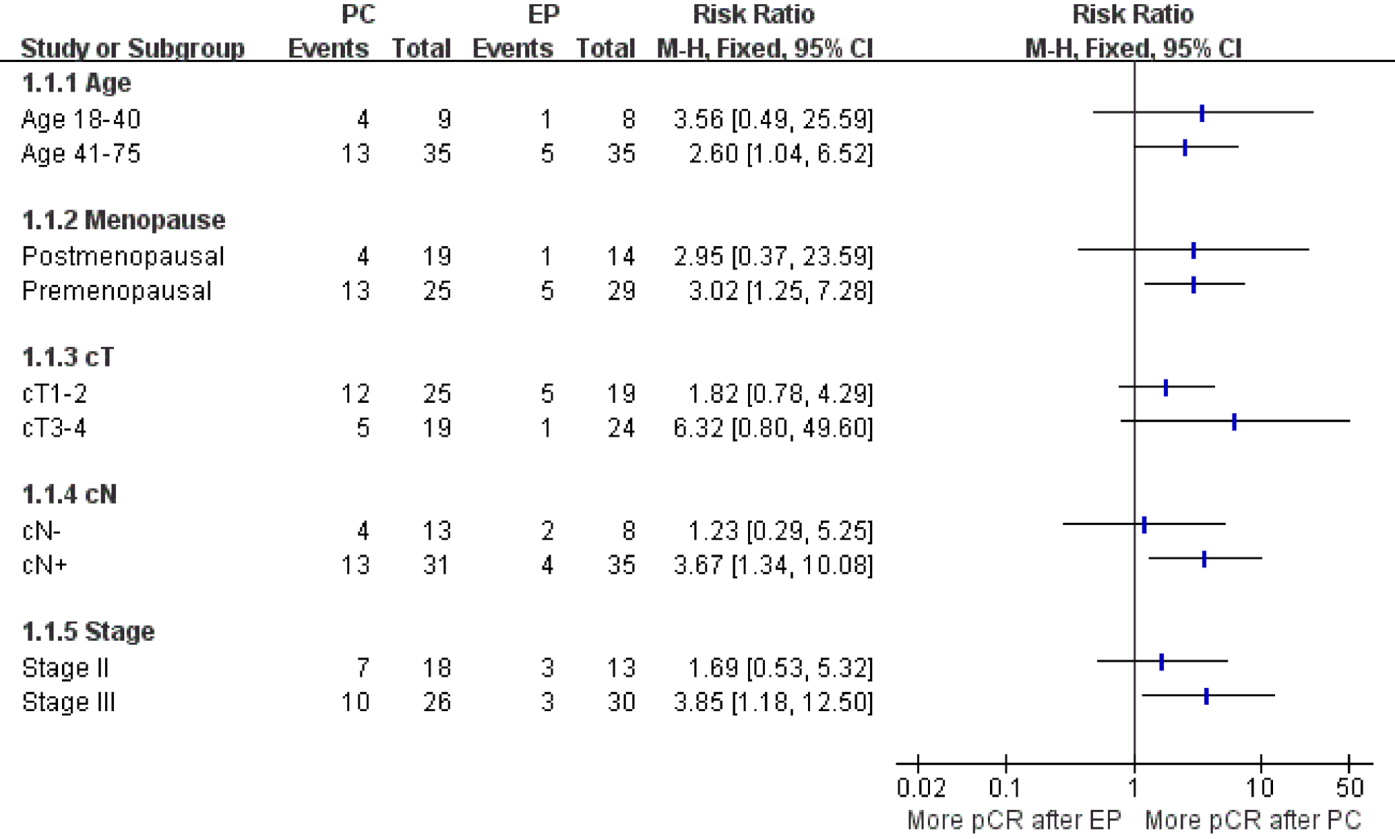
Subgroup analysis in patients achieving pCR after two different treatments PC, paclitaxel plus carboplatin regimen; EP, epirubicin plus paclitaxel regimen; pCR, pathologic complete response.

Figure 3 illustrates the subgroup analysis in patients achieving pCR after two different treatments. The pCR rate for PC was numerically superior in all subgroups, but this difference reached statistical significance only in premenopausal women, clinically evaluated lymph nodes and patients with stage III disease.

### Survival analysis

The cut-off date for survival analysis was November 9^th^, 2015. Median follow-up time was 55.0 months (4.0-105.0 months). Median OS has not been reached. During the study period, a total of 27 relapse events were recorded, 9 in the PC arm and 18 in the EP arm. Most events (88.9%) were observed during the first 3 years after first diagnosis. The most common site of recurrence was lung and lymph node: 4 (44.4%) and 5 (55.6%) patients in the PC arm, and 12 (66.7%) and 7 (38.9%) patients in the EP arm, respectively. The post-recurrence survival for the entire study population was 17.75.6 months, with no significant difference between the two arms (PC arm, 12.13.8 months; EP arm, 18.79.1 months; *P* = 0.445). In the PC arm, there were 7 deaths from any cause, 2 of which died of stroke. Eleven patients died in the EP arm, all of whom were cancer-related deaths.

**Figure 4:**
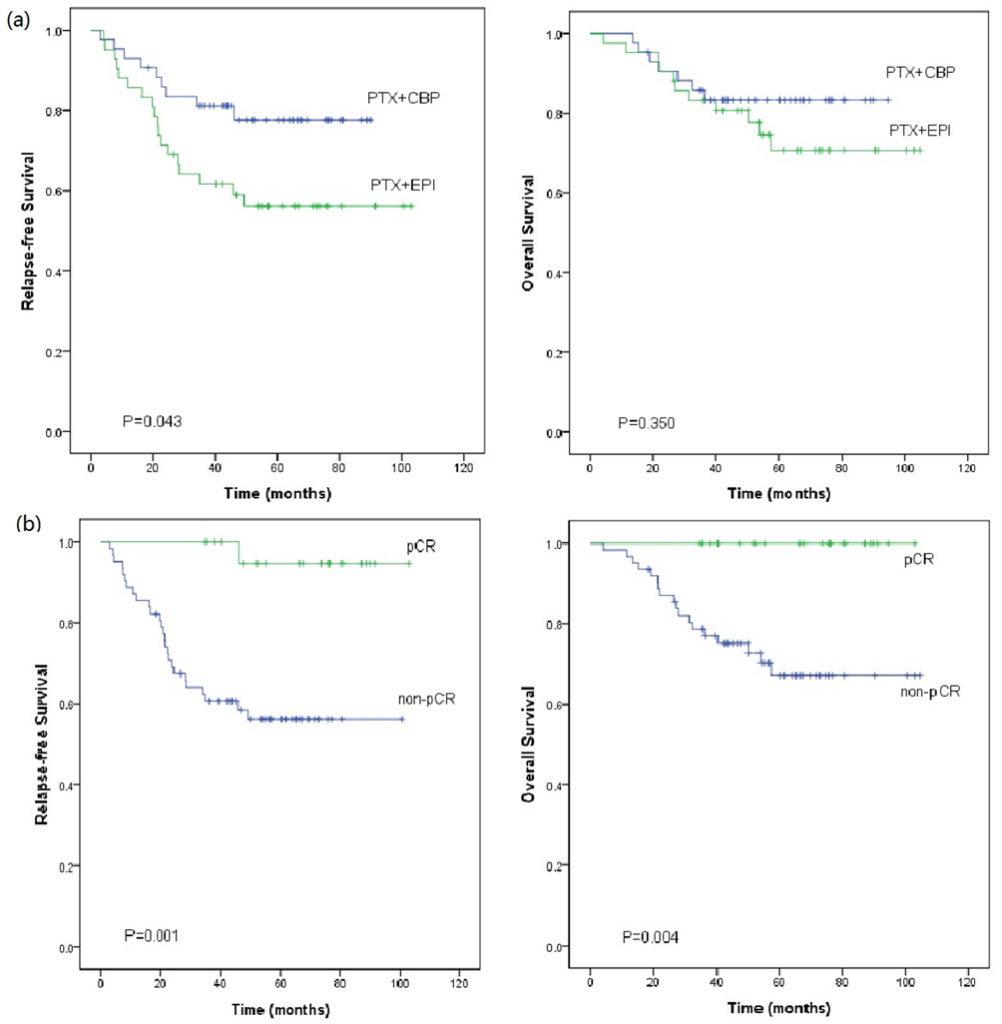
Kaplan-Meier plot of Relapse-free survival (RFS) and overall survival (OS) (a) by different neoadjuvant regimens, and (b) in patients who achieved pathologic complete response (pCR) or not (non-pCR) **a.** Patients in the paclitaxel plus carboplatin (PTX+CBP) arm had significant better RFS when compared to the epirubicin plus paclitaxel (PTX+EPI) arm (*P* = 0.043). The long-term OS of patients in the PTX+CBP arm were almost the same as that of patients in the PTX+EPI arm (*P* = 0.350). **b.** The pCR patients had significantly better RFS (*P* = 0.001) and OS (*P* = 0.004) than the non-pCR patients.

Figure 4 shows the Kaplan-Meier estimates of RFS and OS. Patients in the PC arm had significantly higher RFS rate than that in the EP arm (*P* = 0.043). The 1-, 3-, and 5-year RFS rate were 93.0%, 81.2%, and 77.6% in the PC arm, and 85.7%, 61.6%, and 56.2% in the EP arm, respectively. From another point of view, patients who achieved pCR had significantly improved RFS (*P* = 0.001) compared with those with residual disease after NAC. In 23 patients with pCR, only one of them had disease recurrence. Relapse occurred in the lung 46 months after diagnosis (42 months after surgery), and the patient was still alive after 95.0 months' follow-up. The 5-year RFS rate were 94.7% for patients achieving pCR, and 56.1% for patients with residual disease.

As to the OS, there was no significant difference between the PC regimen and EP regimen (*P* = 0.350). The 1-, 3-, and 5-year OS rate were 100.0%, 85.8%, and 83.3% in the PC arm; and 95.2%, 83.2%, and 70.7% in the EP arm, respectively. Patients who achieved pCR had significantly improved OS (*P* = 0.004) compared with those with residual disease after NAC. The 5-year OS rate were 100.0% for pCR patients, and 67.2% for patients with residual disease, respectively. In patients with residual disease, those who received PC regimens had similar RFS (*P* = 0.217) and OS (*P* = 0.970) compared with those of patients in EP arm.

### NAC safety

Table 2 lists the adverse events. The most common adverse events were neutropenia and vomiting, similar in the two arms. Grade 3/4 neutropenia resulted in treatment delay or dose reduction for 3 patients (6.3%) in the PC arm and 7 patients (15.9%) in the EP arm (*P* = 0.188). Thrombocytopenia occurred more commonly in the PC arm. Grade 3/4 thrombocytopenia was present in 4 patients (8.5%) in the PC arm, resulting in 2 cases of treatment termination. Other adverse events included peripheral neuropathy, ALT/AST elevation, electrocardiographic ST-T changes and myalgia/arthralgia. No death or life-threatening event was recorded during the study.

**Table 2:** Hematological and non-hematological adverse events

	PC arm (*N* =47)		EP arm (*N* =44)		*P* value	
Adverse events	All grade	Grade 3/4	All grade	Grade 3/4	All grade	Grade 3/4
Hematological toxicity						
Neutropenia	43 (91.5%)	34 (72.3%)	37 (84.1%)	28 (63.6%)	0.344	0.500
Thrombocytopenia	17 (36.2%)	4 (8.5)	2 (4.5)	0	0.001	0.118
Non-hematological toxicity						
Vomiting	41 (87.2%)	1 (2.1%)	36 (81.8%)	4 (9.1%)	0.567	0.194
Peripheral neuropathy	19 (40.4%)	0	17 (38.6%)	0	1.000	-
ALT/AST elevation	15 (31.9%)	0	14 (31.8%)	0	0.992	-
Myalgia/arthralgia	10 (21.3%)	0	8 (18.2%)	0	0.715	-
ST-T changes	9 (19.2%)	0	11 (25.0%)	0	0.495	-

Abbreviation: PC, paclitaxel plus carboplatin regimen; EP, epirubicin plus paclitaxel regimen.

Data are n (%) unless stated otherwise.

## DISCUSSION

Two randomized phase II studies, CALGB40603 and GeparSixto, have demonstrated significant increases in pCR rate with the addition of carboplatin to anthracycline-taxane-based NAC in TNBC (54% *vs*. 41%; 57% *vs*. 43%) [8, 9]. In both studies, however, the addition of carboplatin was associated with higher incidence of hematological toxicity, dose adjustments, and treatment discontinuations. In another phase II study (GEICAM/2006-03), it was reported carboplatin did not even improve pCR rate (30% *vs*. 35%) [12]. Furthermore, due to the lack of long-term follow up in these studies, whether higher pCR rates with carboplatin will improve long-term outcomes such as RFS and OS remains unknown.

Our study is the first prospective, randomized, controlled study showing significant improvements in pCR rate and RFS by anthracycline-free platinum-based NAC in TNBC. A long follow-up time also allowed us to report overall survival in these patients.

We found that in the NAC of TNBC patients, the addition of carboplatin to paclitaxel was superior to the regimen of epirubicin plus paclitaxel in terms of pCR rate and RFS. This may due to more frequent deficiencies in the BRCA associated DNA repairing mechanism in TNBC [2, 13, 14]. This was already supported by several studies demonstrating a high level activity of platinum as NAC in BRCA-deficient breast cancers [15, 16]. Telli et al reported that the combination of gemcitabine, carboplatin, and iniparib as NAC in early stage breast cancer resulted in a pCR rate of 33% in wild type BRCA1/2, 47% in BRCA1/2 mutation carriers, and 56% in BRCA1/2 mutation carriers with TNBC [15]. In another study, neoadjuvant cisplatin given every three weeks resulted in a pCR rate of 61 % in TNBC with BRCA1 mutation [16]. Unfortunately, BRCA mutation status was not assessed in this study because of economic and technical conditions at that time. Further comparison of PC and EP as NAC has been planned in breast cancer patients with BRCA-mutations.

Though patients in the PC arm achieved higher rate in pCR and longer RFS than those in EP arm, it should be noted in patients with residual disease after NAC, RFS was similar between the two arms. We may speculate that the improvement in RFS by PC regimen may be directly linked to the increase in pCR rate after NAC. Additionally, in the overall population, patients who obtained pCR had improved long term survival in the study, a finding consistent with previous reports [6]. The RFS inequality might decline because of the switching between PC and EP regimen in the adjuvant setting. Even so, our study has clearly demonstrated the significant improvement of PC over EP in RFS, probably due to the impact of pCR on RFS.

At the time of this report, the median OS has not been reached, which might partly explain the little difference in OS between two arms. Because of the limited sample size and the small number of deaths, our study is unable to detect an OS difference. Other than the duration of follow-up or subsequent treatment upon recurrence, there are still a number of factors that may impact overall survival. Therefore, RFS seems to be a more appropriate indicator than OS here on whether patients have received direct benefit from the treatment.

It is noticed that the pCR rate achieved in our study is lower than previous reports [8, 9, 11]. This may due to the robust triplet regimen, paclitaxel and an anthracycline in addition to carboplatin, used in those studies. Besides a averagely higher tumor stage of patient population, this could also be explained by an every three weeks dosing schedule of paclitaxel used in the study, a dosing schedule later largely replaced by a weekly dosing of paclitaxel because of its higher activity and lower toxicity [8, 17, 18]. When our study started in 2006, the priority of weekly schedule was not definite, and the cost of a weekly schedule of paclitaxel was not covered by patients' medical insurance. That is the reason why we chose 3-weekly schedule of paclitaxel as part of the NAC regimens. Additionally, approximately 95% of our patients were positive in CK5/6 or EGFR biomarkers associated with basal like genotype and poor survival [19-22]. This would also contribute to the lower pCR rate than that reported in other literature.

In summary, this study supports the addition of carboplatin to NAC for TNBC. Anthracycline-free carboplatin-based regimen not only significantly improve pCR rate and RFS but also reduce toxicity, compared to CALGB40603 and GeparSixto studies with concurrent or sequential carboplatin and anthracycline. It is believed carboplatin plus paclitaxel could be an alternative or even a better neoadjuvant chemotherapy option in TNBC. A phase III study adopting dose intense schedule and identifying potential advantageous subgroups with biomarkers such as BRCA mutation has been started to further confirm this hypothesis in the near future.

## MATERIALS AND METHODS

### Study design and assessments

This was a prospective, open label, randomized phase II study. TNBC patients were stratified according to clinical stage (II/III), and then randomized to receive PC (paclitaxel plus carboplatin) or EP (epirubicin plus paclitaxel) regimen as NAC. The primary endpoint was pCR rate, defined as no residual invasive cancer in both excised breast tissue and axillary lymph nodes, or only carcinoma in situ. The secondary endpoints included objective response rate (ORR), RFS, OS and safety.

The clinical evaluation of tumors included physical examination, mammography, ultrasonography of the breast and regional lymph nodes, and breast magnetic resonance imaging (MRI). The clinical or pathological stages were confirmed in accordance with the American Joint Committee on Cancer manual (AJCC, the 6th edition). Clinical responses were assessed every two cycles according to the Response Evaluation Criteria in Solid Tumors (RECIST version 1.0). Adverse effects were defined in accordance with the National Cancer Institute Common Toxicity Criteria for Adverse Events (CTCAE version 3.0). RFS was calculated from the date of randomization to the date of the first local or distant recurrence. OS was defined as the date of randomization to the date of death or last follow-up.

All recruited patients provided written informed consent before treatment as well as verbal consent *via* telephone for the collection of information from their medical record. The protocol was approved by the Institutional Review and Ethics Board of Cancer Institute/Hospital of the Chinese Academy of Medical Sciences. The trial is registered in Clinical Trials. Gov (Trial registration ID: NCT01276769).

### Patient selection and treatment

Major eligibility criteria included: 1) women aged 18-75 years; 2) ECOG score 0-1; 3) pathologically confirmed breast invasive ductal cancer by core needle biopsy, ER/PR/Her-2 negative by immunohistochemistry (IHC), 4) clinical stage IIA-IIIC with NAC indication; 5) measurable lesions; 6) normal cardiac, hepatic and marrow function. Patients were excluded if they had a history of invasive cancer or prior exposure to chemotherapy/radiotherapy.

The PC regimen consisted of paclitaxel 175 mg/m^2^ on day 1 plus carboplatin Area Under the Curve (AUC) = 5 on day 2, both administered *via* intravenous infusion (IV), every 3 weeks for 4-6 cycles. The EP regimen consisted of epirubicin 75 mg/m^2^ on day 1 and paclitaxel 175 mg/m^2^ on day 2, both IV, every 3 weeks for 4-6 cycles.

Patients underwent modified radical mastectomy or breast-conserving surgery within four weeks from the last NAC cycle. Patients who did not complete six cycles of NAC received postoperative chemotherapy. For those who achieved objective response before surgery, additional chemotherapy with the same regime as NAC was administered, with a total of six cycles of perioperative chemotherapy. The rest of patients received alternative adjuvant chemotherapy regimens other than those used in NAC. Postoperative radiation was at the discretion of the treating physicians in accordance with guidelines.

### Tumor tissue assessment

ER and PR were defined as negative when < 10% of nuclei were positively stained in ten high-power fields. Her-2 was considered negative if IHC scoring was 0/1+, or 2+ but FISH negative. Cytokeratin 5/6 (CK5/6), epidermal growth factor receptor (EGFR) and Ki-67 status were assessed by IHC. CK5/6 or EGFR was considered positive if any of the invasive tumors cells showed nuclear staining or membrane staining.

### Sample size justification

The sample size calculation is based on the primary endpoint, i.e. pCR rate. Assuming a rate difference of 0.26 between the two regimens (result of preliminary experiments), a single stage design results in a sample size of at least 42 patients for each arm with a 2-sided type I error rate 0.05 and power 0.8.

### Statistical analysis

All statistical analyses were conducted with SPSS 18.0. Fisher's exact test was used to compare pCR rate, ORR and safety profile between the two regimens. RFS and OS were estimated by the Kaplan-Meier method and compared by log-rank test. Subgroup analysis was performed for the following categorical variables: age, menopause status, tumor size, number of lymph nodes and tumor stage. Risk ratio and its 95% confidence interval were estimated using Mantel-Haenszel method and a forest plot was created using Review Manage 5.2 (http://handbook.cochrane.org/).
